# Indoor Multipath Assisted Angle of Arrival Localization

**DOI:** 10.3390/s17112522

**Published:** 2017-11-02

**Authors:** Stijn Wielandt, Lieven De Strycker

**Affiliations:** Dramco Research Group, Faculty of Engineering Technology, Electronics, KU Leuven, Gebroeders De Smetstraat 1, 9000 Ghent, Belgium; stijn.wielandt@kuleuven.be

**Keywords:** indoor positioning system (IPS), angle of arrival (AoA), multipath assisted localization, NLOS localization, antenna arrays

## Abstract

Indoor radio frequency positioning systems enable a broad range of location aware applications. However, the localization accuracy is often impaired by Non-Line-Of-Sight (NLOS) connections and indoor multipath effects. An interesting evolution in widely deployed communication systems is the transition to multi-antenna devices with beamforming capabilities. These properties form an opportunity for localization methods based on Angle of Arrival (AoA) estimation. This work investigates how multipath propagation can be exploited to enhance the accuracy of AoA localization systems. The presented multipath assisted method resembles a fingerprinting approach, matching an AoA measurement vector to a set of reference vectors. However, reference data is not generated by labor intensive site surveying. Instead, a ray tracer is used, relying on a-priori known floor plan information. The resulting algorithm requires only one fixed receiving antenna array to determine the position of a mobile transmitter in a room. The approach is experimentally evaluated in LOS and NLOS conditions, providing insights in the accuracy and robustness. The measurements are performed in various indoor environments with different hardware configurations. This leads to the conclusion that the proposed system yields a considerable accuracy improvement over common narrowband AoA positioning methods, as well as a reduction of setup efforts in comparison to conventional fingerprinting systems.

## 1. Introduction

In Western society, humans spend approximately 90% of their time indoors [1]. A significant portion is spent in large buildings that might be unfamiliar (e.g., airports, shopping malls, office buildings, etc.). This creates an application domain for indoor localization systems, tracking persons or objects [2]. However, interior environments are characterized by undelimited complex structures, blocking and reflecting signals. The diversity of operating environments and system requirements has led to a multitude of localization systems, most of which can be classified as ‘signals-of-opportunity’ systems, exploiting the existing infrastructure (e.g., Wi-Fi, cellular, lighting, etc.) for positioning purposes. This approach features a low setup cost, but hardware limitations might restrict localization accuracy. In general, the infrastructure consists of fixed reference nodes, transmitting and/or receiving signals. These nodes are often called base stations, anchor nodes or beacons. The device to be localized is commonly denoted as the mobile terminal, mobile node, target or mobile station. Depending on the architecture, this node can be a transmitter as well as a receiver. In these systems, received signal characteristics are measured and converted to parameters that indicate vicinity, distance or direction, eventually leading to a location estimate [3,4,5,6,7].

RF localization systems generally consist of multiple base stations at known locations, and a mobile node with an unknown position [8]. Distinction can be made between proximity, range, angle, and fingerprinting based systems [9,10]. Proximity based (also range-free) systems are considered simple and inexpensive, while offering coarse accuracy. The position of a mobile node in a wireless sensor network is estimated by evaluating which anchor nodes offer a stable connection [11]. Range and angle based positioning schemes follow a geometric approach for localization, relying on respectively measured distances or angles between a mobile node and the anchor nodes. Distance based localization applies lateration methods to estimate the location of a node. In RF systems, distances can be obtained by measuring the Received Signal Strength (RSS) or Time of Flight (ToF), relying on respectively the declining signal strength or the increasing travel time over distance. In angulation based geometric localization systems, signal directions are represented as straight lines. The intersection of these lines indicates the location of the node. Angular systems require anchor nodes and/or the mobile node to be equipped with multiple antennas in order to estimate signal directions. Direction of Arrival (DoA) systems measure the directions of received signals at the base station side. In a 3D environment, these directions are defined by an azimuth and elevation angle. In a simplified approach for 2D operation, the term ‘Angle of Arrival’ (AoA) is used, estimating angles in a single plane. However, this 2D simplification represents a possible source of positioning errors, since the AoA represents only a single broadside angle [8]. The broadside angle only equals the azimuth angle when transmitter and receiver are in the same plane (i.e., the elevation angle is zero).

An important drawback of RF positioning systems lies in their susceptibility to indoor multipath effects such as reflections, scattering, diffraction, refraction and absorption, especially in NLOS conditions when shadowing occurs [10,12,13]. In ToF and DoA systems, these changes of the propagation path result in erroneous measurements. In RSS systems, the multipath effects lead to fluctuations of the signal strength, complicating localization efforts. However, the RSS approach is the most commonly used indoor localization method due to the generally sufficient performance and the omnipresence of wireless communication systems, making the deployment of dedicated hardware for localization unnecessary. The multipath phenomena are usually considered as random events and their negative influence is mostly reduced by averaging, filtering or redundancy. Another way to deal with multipath propagation in real-world situations consists of fingerprinting. This technique is mostly applied in RSS systems and takes the effects of the environment on signal characteristics into account, limiting localization errors [3]. This method consists of an offline phase, in which a reference dataset is built from surveyed signal characteristics at known positions. In the online phase, measurement data is matched to the reference dataset, leading to an estimated position. A major drawback of fingerprinting lies in the labor intensive offline survey phase, contributing to a high deployment cost. Also, the technique is susceptible to changes in the environment, which alter the propagation channel.

The variety of indoor positioning techniques indicates that no universal approach exists for all requirements. Even though RSS systems provide a viable solution for many present-day applications, the combination of challenging requirements, current system limitations and continuous innovations in RF communication systems form an impulse for new research contributions. Over the past years, widely deployed RF communication systems have gained Multiple Input Multiple Output (MIMO) properties (e.g., 802.11ac [14], cellular systems [15], etc.). This multi antenna approach will evolve even further to Massive MIMO (MaMIMO) solutions in next generation radio systems [16]. In the first place, these multi antenna systems allow more accurate RSS readings, since small scale fading effects can be overcome. In the short term, this can lead to an improvement of existing RSS localization systems. However, the narrowband antenna arrays in these communication systems could also be used for measuring angular signal parameters. While AoA is currently not a widely used localization technique, it could provide accuracy improvements or cost reductions in future positioning systems. This work investigates a new indoor RF localization approach, relying on the angular (AoA) information of multipath components, extracted from narrowband antenna arrays. The array based solution possibly allows a low cost implementation due to the potential ‘signals-of-opportunity’ approach and the massive spread of compatible mobile devices. The intended goals consist of an improvement of accuracy and robustness, while simplifying the deployment with respect to comparable systems. However, this work should be seen as a first experimental phase to test the feasibility of the approach. Further research steps are required to obtain a practically deployable system for real-time localization of multiple mobile nodes.

As previously explained, the multipath propagation channel is usually considered as an error source because it disrupts the measurement of LOS signal characteristics. Nevertheless, the proposed localization method aims to exploit this propagation phenomenon to obtain extra spatial information. Therefore, a ray tracing algorithm is used to generate valuable multipath information based on an a-priori known floor plan. The simulated multipath data is then used in a localization algorithm. With this ‘multipath assisted’ approach, even NLOS connections can contribute to the positioning accuracy instead of causing errors. The envisioned localization method is primarily aimed at LBS applications that already rely on RF infrastructure (cfr. contemporary RSS systems). The target improvements include an increased accuracy and NLOS robustness, a reduced number of anchor nodes, and reduced setup efforts in comparison to conventional fingerprinting systems. The reduction of setup efforts is achieved by replacing the labor-intensive site survey phase with multipath calculations. In a finalized localization system, this should result in a simplified deployment phase in large scale complex environments. This even opens doors for new applications, such as temporary installations at events or temporary constructions.

The localization performance is verified by measurements with lab equipment in real-world environments. Because a finished setup is not aspired, the algorithms for processing the measurement data are implemented in a Matlab^®^ framework for testing. The goal of the experiments is to evaluate the pure performance of the multipath assisted algorithm. Therefore, the software does not implement travelled path tracking, dead reckoning, Kalman filtering, particle filters, or any other post processing algorithms for real-time tracking.

The remainder of this text is structured as follows: Section 2 presents the related work and state-of-the-art. Section 3 describes the proposed localization method. This section starts with a general overview of system requirements and assumptions, followed by a theoretical overview of the localization algorithm, focusing on AoA estimation and ray tracing techniques. The section concludes with a presentation of evaluation criteria for assessing algorithm performance. Section 4 contains an extensive discussion of experimental results in different test scenarios with various hardware parameters. Section 5 discusses the research results with respect to related research. Section 6 recapitulates the most important realizations and findings, followed by a discussion of future research opportunities in the field.

## 2. Related Work

In related research, multipath signals are mostly treated as error sources that should be detected and mitigated. Most NLOS handling algorithms rely on statistical methods. As NLOS signals generally do not match the expected LOS characteristics, these situations can be statistically detected and treated as outliers. In the time domain, this approach only works for occasional NLOS connections [17]. An approach in the spatial domain is proposed in [18], using redundant anchor nodes. If a NLOS connection is detected, it is excluded from the positioning algorithm. A similar method is used in [19], performing triangulation in a cellular network with only the two most probable LOS connections. Garcia et al. propose a narrowband AoA localization system for outdoor use in MaMIMO communication systems with multiple base stations [20]. The NLOS problem is tackled by a direct localization approach called ‘Direct Source Localization’ (DiSouL): instead of performing triangulation with the strongest signals, all received multipath components are processed by a ‘fusion center’ that determines the LOS directions, leading to an estimated position through triangulation. For NLOS situations, this means that very weak LOS signals can be used in a triangulation algorithm, even when stronger NLOS components exist. In an outdoor scenario with synthetic data, the system exhibits superior performance over the classical triangulation approach, achieving sub-meter accuracy in favorable conditions (SNR over 10dB, over 80 array elements). Fang et al. present an RSS fingerprinting system that performs a multipath effect reduction on measurement results before estimating the position of a user [21]. The technique is demonstrated in a 24.6 m × 17.6 m environment, presenting a significant improvement of localization accuracy over a standard RSS fingerprinting approach. Absolute localization errors heavily depend on the number of anchor nodes: mean errors range from 6.4 m to 0.5 m for respectively 1 to 8 anchor nodes.

Most indoor localization systems consider multipath components as undesirable because they introduce localization errors. However, these components contain spatial information that can be exploited, a feature that is explored in multipath assisted localization systems. Meissner et al. developed a system for multipath assisted indoor navigation and tracking (MINT) [7]. Ranging measurements are performed with a ToA UWB system with a 2 GHz bandwidth around a 7 GHz or 8 GHz center frequency. The system measures the distances of direct and reflected signal paths between the anchor node and the mobile node. With the help of ray tracing algorithms and a-priori known information of the room geometry, the location of the mobile node can be retrieved. The required availability of floor plan information should not be considered as an insuperable restriction, as most systems already use a floor plan for visualizing the localization outcome. For the simulation of reflected multipath components, calculations rely on the image method: so called ‘virtual anchor points’ are created as mirror images of the physical anchor node with respect to the walls [22,23]. The available measurement data and ray tracing algorithms enable multilateration of direct and reflected signals, leading to an estimated position of the mobile device. The result is a system that exploits multipath information, increasing localization accuracy and overcoming NLOS problems. Furthermore, the system can operate with a single anchor node because multilateration can be performed with multiple signal components. The system performance was demonstrated in a 4.5 m × 5.5 m room, showing <0.20m localization errors for 95% of the tested positions [24]. In a 6 m × 8.5 m room, 95th percentile localization errors of 0.08 m to 0.20 m were reported, depending on the accuracy of the room geometry model [25]. Another configuration was evaluated in a setup for tracking indoor pedestrian movements. Therefore the system was expanded with a motion model for pedestrians, correcting localization imperfections. In this setup of a 25 m × 25 m room with a single anchor node, <0.70m localization errors are achieved for 95% of the tested positions [26]. Operation in NLOS conditions was claimed, but no test results were reported. A similar system is presented by Van De Velde et al. [27,28]. The proposed ‘cooperative UWB positioning indoors’ (CUPID) algorithm relies on the same principle of multipath ranging and ray tracing based multilateration. The difference lies in the determination of multipath weights, here relying on a cooperative algorithm that requires multiple mobile users. In a 10 m × 25 m room with LOS connections of at least three cooperating mobile nodes, a 95th percentile localization error of 0.70 m was reported. The results of these multipath assisted UWB ranging systems demonstrate sub-meter and sometimes even centimeter accuracy for a single anchor node positioning system. These exceptional results can be attributed to the favorable UWB signal characteristics. However, the UWB approach is not compatible with narrowband communication systems, preventing a merge of these techniques with contemporary communication technologies.

As explained before, fingerprinting techniques can be used to account for the environmental effects on signal characteristics. This means that multipath effects are included in the localization process, making fingerprinting systems a type of multipath assisted localization system. Most fingerprinting systems use omnidirectional RSS data of multiple anchor nodes because of the standard availability in wireless communication systems, requiring no further hardware investments. However, some solutions with a single anchor node have been proposed, performing RSS fingerprinting for different directions of arrival. This uncommon method of DoA localization relies on measurements with multiple antennas. In [29,30] an anchor node with six directional antennas is proposed, using antenna switching to measure signal characteristics in different directions. The fingerprinting localization algorithm yields an average localization error of 2.32 m in a 7.20 m × 8.00 m room with LOS conditions. Another example can be found in [31], describing a 1 + 12 elements parasitic array for measuring RSS values. The reported errors in an indoor 4.5 m × 4.5 m LOS area exhibit mean values ranging from 1.66 m to 1.86 m and median values of 1.12 m. More accurate results can be obtained by equipping both the anchor node and the mobile node with an antenna array, an approach that is presented in [32]. In an ideal 4 m × 5 m area, average localization errors below 0.2 m were achieved. In [33], a Massive MIMO fingerprinting system is proposed for outdoor use. Localization of a mobile device is performed with a single base station, which is equipped with 36 to 100 antennas. Instead of performing beamforming, the algorithm uses an RSS vector containing channel hardened RSS values for each antenna of the base station (i.e., small scale fading is reduced). The base station consists of a large 50 m × 50 m antenna array, performing localization in a 150 m × 200 m area. It should be noted that the size of the array is comparable to the size of the testing area, justifying the RSS vector approach. In all system configurations, >30m RMS localization errors are reported.

Another research domain in the context of indoor positioning systems focuses on the simplification of the deployment phase. So-called Easy to Deploy Indoor Positioning Systems (EDIPS) aim for reduced setup times by using existing infrastructure and simplifying setup efforts. In [34,35], WiFi infrastructure is used for RSS measurements. The localization step resembles a fingerprinting approach, however the fingerprints are obtained in calculations instead of a labor intensive survey phase. Further simplifications are performed by eliminating the influences of walls and other obstacles. The described system uses six anchor nodes and can reportedly be deployed on a $1320\;m^{2}$ building floor in 12 to 15 min. These systems usually do not aspire high accuracy localization, resulting in room-level accuracy and reported peak localization errors up to 31 m. A similar WiFi based system is described in [36], however lateration algorithms are used instead of calculated fingerprints, resulting in a similar room-level accuracy.

## 3. Methods

### 3.1. Assumptions, Boundary Conditions and Requirements

As demonstrated in literature, multipath components contain spatial information that can be exploited by localization systems. This paper explores a new localization approach, leveraging on these ideas. Before explaining the core architecture, the assumptions and boundary conditions are discussed. The technique is centered around AoA measurements to observe the multipath environment. A narrowband AoA approach with antenna arrays is selected for its conformity with contemporary (Massive) MIMO systems, enabling a future implementation in communication systems (e.g., 802.11ac [14] and cellular systems [15], etc.). This property resembles the approach of widespread RSS localization systems, relying on communication networks. From this point of view, the 2.4 GHz ISM band is selected for all experiments. A network based approach is proposed, offloading all processing to the server side. This relaxes the computational requirements for mobile devices, making the technology accessible for sensor networks. Considering the practical implementation of the system, only single room setups with a single mobile node are currently evaluated. This ensures maximum control of environmental parameters, resulting in straight-forward and consistent LOS and NLOS testing. Furthermore, results will be comparable to the related work, which mostly applies a similar approach. With respect to anchor nodes, multiple assumptions and requirements are put forward. For AoA measurements, uniform linear arrays are proposed. The symmetrical structure and a uniform λ/2 inter-element spacing allows AoA processing techniques like forward-backward averaging and spatial smoothing, while maintaining a field of view of $180^{\circ }$. This allows a 2D simplification of the localization problem, assuming elevation angles of $0^{\circ }$. A 2D floor plan is sufficient in this respect, and mobile nodes and anchors are always placed in the same horizontal plane (i.e., at the same height). For the placement of anchor nodes, only lateral positions are considered, close to the walls of the room. From a user point of view, this means that no inconvenient infrastructure is required in the middle of the room. From a technical point of view, this design choice solves the problem with ULAs not being able to distinguish the frontside from the backside of the array [37]. By placing the array against a wall, signals from the backside can be eliminated, so all estimated AoA values can be considered frontal. Furthermore, the system should be able to operate with a single anchor node. As demonstrated in [29,31], a single antenna array can be used for positioning by measuring multipath propagation. However, the addition of extra nodes should be straight-forward, resulting in a flexible system. An important requirement is a straight-forward and easy setup phase, which does not only speed up the installation of a system, but also the development and evaluation. It is clear that a classical fingerprinting approach does not meet these requirements. Therefore, a multipath assisted algorithm is proposed, estimating a position based on AoA measurements.

### 3.2. Localization Algorithm

**Figure 1:**
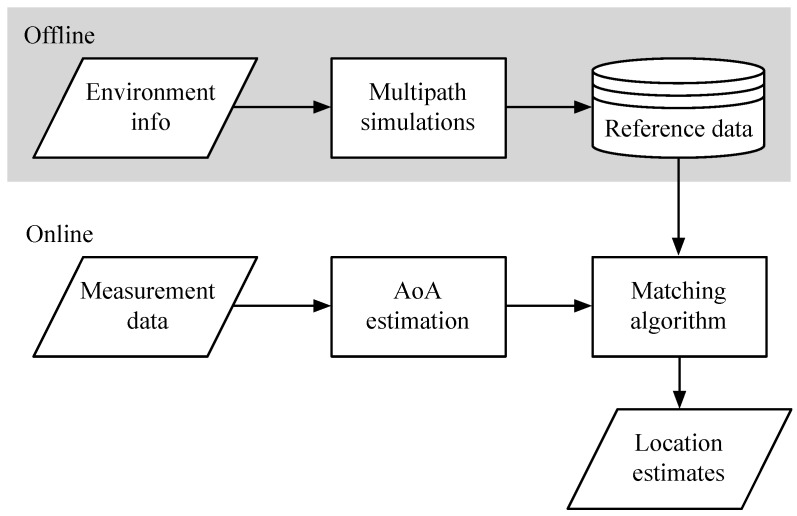
Architecture of the localization process with a single anchor node.

The outline of the envisioned localization algorithm for a single anchor node is visualized in Figure 1. The overall structure resembles a fingerprinting approach with an online and offline phase. However, no labor intensive site surveying is required in the training phase, but multipath calculations are performed instead. These calculations are performed in the offline phase in order to alleviate the computational load during localization (i.e., the online phase). Only the fixed infrastructure is taken into account, ignoring movable objects like furniture. As a result, only the guaranteed multipath components are included, reducing the impact of changes in the environment. This approach requires only a basic floor plan without details, facilitating the setup of the system. The calculations are performed along a fine grid of training positions $p_{i}=\left(x_{i},y_{i}\right)$ in the room. For each grid point, the propagation path is simulated and stored as a fingerprint $f_{i}$. The resulting training vector T contains multipath AoA information for every position in the room.

(1) $$T=\left\{\left(f_{1},p_{1}\right),\left(f_{2},p_{2}\right),\ldots ,\left(f_{N_{f}},p_{N_{f}}\right)\right\}$$

The online phase always starts with measurement data from an antenna array (i.e., phase and amplitude information of each channel). This data is processed by AoA estimation algorithms, forming a representation of the multipath environment as vector m. A pattern matching algorithm is used to calculate the resemblance between the measurement vector m and the simulated training data. The result can be represented as a ‘Spatial Probability Density Function’ (SPDF), visualizing the probability of the transmitter location for each position $p_{i}$ in the room. The position with the highest probability yields the estimated location. When multiple anchor nodes are used, the localization process is repeated for each anchor node, resulting in multiple SPDFs. These results can be combined, as discussed in Section 3.7. The same approach can be applied for combining localization results of various signal characteristics.

### 3.3. Measurement Vectors

**Figure 2:**
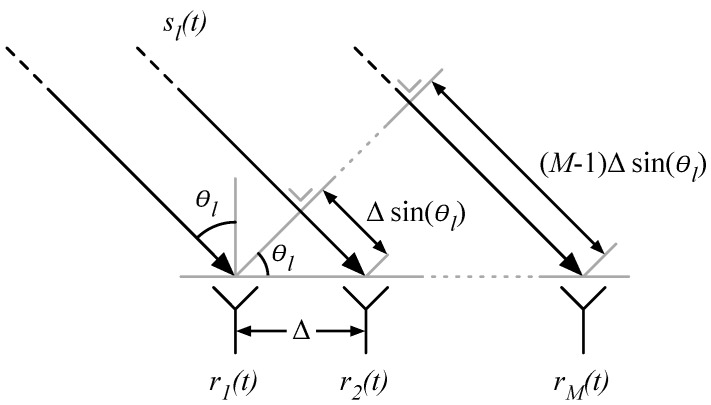
ULA system model.

The measurement vector m is the result of an AoA estimation technique, transforming the measured phase and amplitude information of an antenna array to the angular domain [10,37,38]. The schematic representation in Figure 2 depicts *M* antennas with an inter-element distance Δ. *L* uncorrelated wavefronts $s_{l}\left(t\right)$ ($l\in \left\{1,\ldots ,\;L\right\}$) impinge on the array under an angle $\theta_{l}$ ($\theta_{l}\in \;\left[-90^{\circ },90^{\circ }\right]$, i.e., the angle of arrival), resulting in the received signals $r_{m}\left(t\right)$ ($m\in \left\{1,\ldots ,M\right\}$). In the further theoretical discussion planar wavefronts are assumed, so signals travel in parallel. This condition is fulfilled when signal sources are located in the far field, as expressed by Equation (2). This rule-of-thumb expression depends on the array size Δ(M−1) and the wavelength λ. Furthermore, the medium is assumed to be isotropic and linear, allowing linear superpositions of signals.

(2) $$d>\frac{2{\left[\Delta \left(M-1\right)\right]}^{2}}{\lambda }$$

When a wavefront impinges on the array, it arrives with a time delay $\Delta \sin(\theta_{l})$ at each consecutive antenna, resulting in phase differences $\mu_{l}$, as expressed by Equation (3). $f_{c}$ represents the carrier frequency of the system, which is assumed to be smallband. This means that the phase and amplitude of $s_{l}\left(t\right)$ changes slowly with respect to $\Delta \sin(\theta_{l})$.

(3) $$\mu_{l}=\frac{2\pi f_{c}}{c}\Delta \sin\left(\theta_{l}\right)=\frac{2\pi }{\lambda }\Delta \sin\left(\theta_{l}\right)$$

The phase differences $\mu_{l}$ are used to determine the values of $\theta_{l}$. This requires a one-on-one relationship between $\mu_{l}$ and $\theta_{l}$, which can be expressed as $\left|\mu_{l}\right|\leq \pi$. Substituting this condition in Equation (3) results in the requirement Δ≤λ/2. Usually, the λ/2 spacing is adopted, as smaller values stimulate practical problems like antenna coupling and small scale fading. A larger spacing results in phase ambiguities, which manifests as grating lobes in the array response pattern. Furthermore, it must be noted that $\theta_{l}\in \left\{-90,-89,\ldots ,0,\ldots ,89,90\right\}$ results in the same phase differences $\mu_{l}$ as $\theta_{l}\in \left\{-90,-91,\ldots ,180,\ldots ,91,90\right\}$. This means that a ULA cannot distinguish the frontside from the backside of the array.

The received signal at antenna element *m* is described in Equation (4), with $H_{m}\left(f_{c},\theta_{l}\right)$ representing the *m*-th antenna response and $\eta_{m}\left(t\right)$ being the noise at element *m*. The noise is assumed to be white and gaussian with a zero mean and variance $\sigma_{\eta }^{2}$ (i.e., AWGN). It is not correlated with $s_{l}\left(t\right)$ and there is no noise correlation between array elements. These properties form an approximation of the noise in the propagation channel and in the separated array channels. The noise is assumed to follow a natural physical behaviour (e.g., thermal noise) without any interfering non-AWGN signal sources. This enables a further mathematical analysis and distinction of signal components. Of course, any deviation from these assumptions in practical setups (e.g., coupling between array channels, interfering communication systems, etc.) will result in a reduced performance.

(4) $$r_{m}\left(t\right)=\sum_{l=1}^{L}H_{m}\left(f_{c},\theta_{l}\right)s_{l}\left(t\right)e^{-j\left(m-1\right)\mu_{l}}+\eta_{m}\left(t\right)$$

$H_{m}\left(f_{c},\theta_{l}\right)e^{-j\left(m-1\right)\mu_{l}}$ is defined as the *m*-th element of the array steering vector a(θ). In most configurations all array elements are equal, resulting in a single antenna response $H(f_{c},\theta )$, as expressed in Equation (5). The array outputs for *L* signals $s_{l}\left(t\right)$ are expressed in Equation (6), relying on the linear nature of the medium.

(5) $$a\left(\theta_{l}\right)=H\left(f_{c},\theta_{l}\right){\left[1\;\;e^{-j\mu_{l}}\;\;\ldots \;\;e^{-j\left(M-1\right)\mu_{l}}\right]}^{T}\;{\;}_{H\left(f_{c},\theta \right)=H_{1}\left(f_{c},\theta \right)=\ldots =H_{M}\left(f_{c},\theta \right)}$$

(6) $$\left[\begin{array}{c} r_{1}\left(t\right)\\ r_{2}\left(t\right)\\ \vdots \\ r_{M}\left(t\right) \end{array}\right]=\left[a\left(\theta_{1}\right)\;\;a\left(\theta_{2}\right)\;\;\ldots \;\;a\left(\theta_{L}\right)\right]\left[\begin{array}{c} s_{1}\left(t\right)\\ s_{2}\left(t\right)\\ \vdots \\ s_{L}\left(t\right) \end{array}\right]+\left[\begin{array}{c} \eta_{1}\left(t\right)\\ \eta_{2}\left(t\right)\\ \vdots \\ \eta_{M}\left(t\right) \end{array}\right]$$

In matrix notation, this is reduced to Equation (7), with A denoting the array manifold, i.e., a collection of *L* steering vectors, defining the array response to all impinging wavefronts.

(7) r(t)=As(t)+η(t)

The received signals r(t) contain uncorrelated noise and correlated signal components, originating from the same signal sources. This property can be exploited for the extraction of AoA information. Therefore, the spatial covariance matrix R is introduced, as expressed in Equation (8). E{} denotes the statistical expectation operator, while S and $R_{\eta }$ represent the spatial correlation matrices of s(t) and η(t). In real-world systems, R will always be calculated from multiple array measurements over time, resulting in an estimated spatial covariance matrix $\tilde{R}$.

(8) $$\begin{array}{c} R=E\left\{r\left(t\right)r{\left(t\right)}^{H}\right\}={\mathrm{ASA}}^{H}+R_{\eta }={\mathrm{ASA}}^{H}+\sigma_{\eta }^{2}I_{L} \end{array}$$

In order to estimate the $\theta_{l}$ values, different AoA estimation algorithms are available. In non-parametric algorithms, sometimes referred to as quadratic algorithms, no assumptions are made about the statistical properties of the signals. The AoA estimation is performed by steering the beam electronically over all directions and measuring the output power of the beamformer. This results in a ‘spatial spectrum’, which indicates the received power P(θ) as a function of the steering direction θ. The peaks in this spectrum indicate the estimated values of $\theta_{l}$. Steering the beam of the array is done by linearly combining all antenna signals with a complex weight vector w, as expressed in Equation (9). In order to determine the values of the weight vector, knowledge of the array steering vectors a(θ) is required.

(9) $$\begin{array}{c} P\left(\theta \right)=w^{H}\tilde{R}w \end{array}$$

In parametric estimators, assumptions are made about the statistical characteristics of the received signals. A part of the parametric algorithms can be classified as subspace-based or super-resolution estimators (e.g., MUSIC, ESPRIT). However, these algorithms are not considered in this research because they only deliver discrete AoA values instead of a spatial spectrum. Furthermore, the performance heavily degrades in multipath environments because of reflected signals. Previous research has indicated that the non-parametric MVDR (Minimum Variance Distortionless Response) algorithm is a preferable AoA estimator in this system [39].

#### 3.3.1. MVDR

The Minimum Variance Distortionless Response (MVDR) algorithm, also known as the Capon beamformer, does not follow a standard beamforming approach. The focus of this algorithm is not on power maximization in the looking direction, but on minimization of average power ($E\left\{{\left|y(t)\right|}^{2}\right\}$) while maintaining unity response in the looking direction ($w^{H}a\left(\theta \right)=1$) [40]. The resulting weight vector w is described in Equation (10), while the spatial spectrum $P_{MVDR}\left(\theta \right)$ is expressed by Equation (11).

(10) $$w_{MVDR}\left(\theta \right)=\frac{{\tilde{R}}^{-1}a\left(\theta \right)}{a{\left(\theta \right)}^{H}{\tilde{R}}^{-1}a\left(\theta \right)}$$

(11) $$P_{MVDR}\left(\theta \right)=\frac{1}{a{\left(\theta \right)}^{H}{\tilde{R}}^{-1}a\left(\theta \right)}$$

This algorithm achieves superior AoA estimation performance because the sidelobes of the beamformer are reduced. This assures better performance when multiple signals impinge on the array, but it comes at the cost of a higher computational load. In spite of the increased overall performance, this beamforming algorithm still underperforms when signal sources are correlated. An interesting remark with respect to AoA estimation algorithms is their resemblance to frequency spectral estimators [41,42]. In this analogy, $\mu_{l}$ is called the ‘spatial frequency’ and the inter-element spacing requirement Δ≤λ/2 can be linked to the Nyquist sampling theorem in the time domain. More interestingly, the weight vectors w can be considered as filter weights in the spatial domain, in analogy to Finite Impulse Response (FIR) filter coefficients in the time domain. In the MVDR algorithm, filter weights are determined by $\tilde{R}$. This means that the shape of the filter depends on the received signals (cfr. adaptive filters).

#### 3.3.2. Signal Decorrelation

The performance of AoA estimation algorithms is negatively impacted by correlated signal sources. In order to estimate the angles of *L* impinging signals, R is required to have rank *L*. This means that S should be diagonal and singular, a condition that is only fulfilled when the *L* signal sources are uncorrelated. This condition deserves particular attention in multipath environments, as multiple signals originate from the same source and are consequently correlated. A possible solution consists of signal decorrelation techniques like Forward-Backward Averaging (FBA) or Spatial Smoothing.

#### 3.3.3. Forward-Backward Averaging

The FBA preprocessing technique is only applicable in symmetrical antenna arrays (a ULA for example). This method relies on the fact that steering vectors remain the same when their order is reversed and their values are complex conjugated. Using this property, a backward spatial covariance matrix can be defined as in Equation (12), with $\Pi_{M}$ denoting an M×M exchange matrix (anti-diagonal matrix of ones).

(12) $$R_{\mathrm{back}}=\Pi_{M}R^{*}\Pi_{M}$$

Forward-backward averaging is achieved by averaging the spatial covariance matrix with its backward counterpart, as expressed by Equation (13). In case of correlated signals, this manipulation achieves one decorrelation, resulting in an increased rank of ${\tilde{R}}_{\mathrm{fb}}$.

(13) $$R_{\mathrm{fb}}=\frac{1}{2}\left(R+R_{\mathrm{back}}\right)$$

#### 3.3.4. Spatial Smoothing

Another solution consists of spatial smoothing, a preprocessing technique for signal decorrelation that divides the array into $K_{\mathrm{ss}}+1$ subarrays containing $M_{\mathrm{sub}}=M-K_{\mathrm{ss}}$ elements, each with a separate spatial covariance matrix $R_{\mathrm{sub},k}$. A new spatially smoothed spatial covariance matrix $R_{\mathrm{ss}}$ can be obtained by averaging the spatial correlation matrices of the subarrays as presented in Equation (14). The result is a spatial covariance matrix $R_{\mathrm{ss}}$ with rank *L*, assuming that the array is equipped with a sufficient number of elements *M*, as supported by Equation (15).

(14) $$R_{\mathrm{ss}}=\frac{1}{K_{\mathrm{ss}}+1}\sum_{k=1}^{K_{\mathrm{ss}}+1}R_{\mathrm{sub},k}$$

(15) $$\begin{array}{cc} M & \geq L+K_{\mathrm{ss}}+1 \end{array}$$

In the case of correlated signals, spatial smoothing enhances the rank of the spatial covariance matrix by $K_{\mathrm{ss}}$, but it decreases the array aperture to $M_{\mathrm{sub}}$, limiting the number of detectable signals. Therefore, the amount of spatial smoothing operations $K_{\mathrm{ss}}$ will always be a trade-off between the array aperture (the number of detectable signals) and the number of signal decorrelations. This contrasts with the FBA technique, which does not affect the array aperture, but performs only one signal decorrelation.

#### 3.3.5. MVDR Measurement Vectors $m_{\mathrm{MVDR}}$ and $m_{\mathrm{LOS}}$

**Figure 3:**
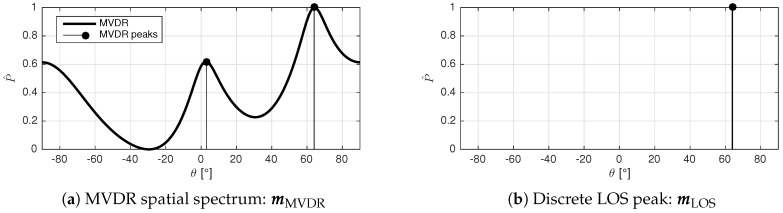
Example measurement vectors.

The MVDR measurement vector $m_{\mathrm{MVDR}}$ represents a standard MVDR spatial spectrum $P_{\mathrm{MVDR}}\left(\theta \right)$, which can be matched to the fingerprint vectors. The peaks in the MVDR spectrum indicate the AoA values. Forward backward averaging is applied in all situations, while the amount of spatial smoothing $K_{\mathrm{ss}}$ is selectable. An example of an MVDR based measurement vector is depicted in Figure 3.

Classical AoA localization systems rely solely on the LOS direction, which is considered the strongest peak in the spatial spectrum. This discrete angular value represents a benchmark measurement vector $m_{\mathrm{LOS}}$ that can be used to compare the performance of the classical AoA approach with the multipath assisted approach. An example of a benchmark measurement vector is depicted in Figure 3.

### 3.4. Reference Vectors

In the offline phase of the localization system, channel simulations are performed to generate a training set of AoA data. Multiple approaches can be followed to predict signal propagation, as discussed in literature [43,44]. This work relies on ray tracing, a technique that performs multipath simulations based on Geometric Optics (GO) [45]. The energy of the electromagnetic waves is assumed to travel through infinitesimally small tubes called ‘rays’. These rays indicate the travel direction of the waves in a straight line, normal to the plane of equal signal power. In order to calculate rays, the ‘image method’ is applied [46,47]. By mirroring the complete geometry of the environment against each possibly reflecting surface, images are created with virtual transmitters or receivers. A basic framework was developed to calculate the various paths that can be followed from transmitter to receiver. The starting point is a two-dimensional map of a rectangular room with one antenna array and one mobile transmitter. This basic approach was considered sufficient given the early research stage, limiting tests to rectangular rooms. Furthermore, it makes the image method particularly appropriate for generating the required fingerprints.

**Figure 4:**
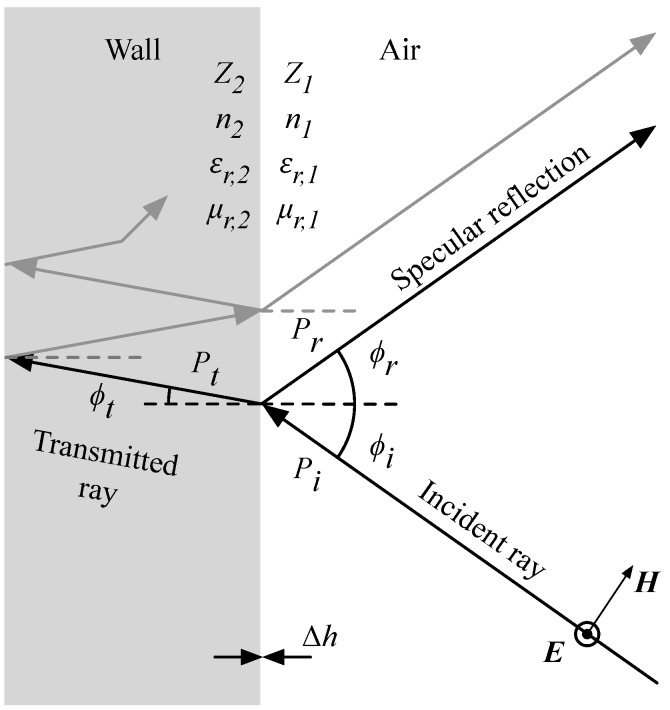
Multipath effect at a smooth and large material boundary.

The multipath calculator only considers the LOS connection and specular reflections up to a given order. Diffracted and scattered components are not considered because they do not contain valuable spatial information on the location of the transmitter, as scattering and diffraction cause unknown changes in the directions of the rays [24]. Furthermore, previous research has indicated that these components generally carry a significantly lower energy than the line-of-sight (LOS) connection or specular reflections in indoor environments for the considered frequency domain [48,49,50,51]. At the large material boundaries, the power of the incident wave $P_{i}$ is split into a reflected component $P_{r}$ and a transmitted component $P_{t}$, as depicted in Figure 4. Specular reflections only occur at sufficiently smooth material boundaries. This condition is met when the boundary imperfections (Δh in m) meet the Rayleigh criterion of Equation (16) [52]. The grazing angle (i.e., the angle of the impinging rays) is denoted as $\phi_{i}$. For 2.4 GHz signals, this means that Δh<0.0156m, a criterion that can be assumed valid for most indoor walls.

(16) $$\Delta h<\frac{\lambda }{8\cos(\phi_{i})}$$

Specular reflections are calculated according to the Fresnel formulas, with incident and reflected angles being equal to each other ($\phi_{i}=\phi_{r}$) [52,53,54]. This property is the foundation of multipath assisted systems: because of this predictability, signal paths can be traced, enabling the calculation of a transmitter position, be it in the time domain or angular domain. In the simulated environment, a vertically polarized antenna is assumed for the transmitter (e.g., a half wavelength vertically oriented dipole). This means that the considered reflections against the walls are transverse electric: the electric field vector (E) is normal to the plane of incidence (i.e., the plane that contains the incident and reflected rays). In this case, the reflection coefficient $\Gamma_{⊥}$ can be calculated as in Equation (18), which is a function of $\phi_{i}$ and the field impedances of medium 1 and 2 ($Z_{1}$ and $Z_{2}$) [54]. These quantities are a function of wave polarization, grazing angle $\phi_{i}$, the relative permittivity and permeability of air ($\varepsilon_{r,1}$ and $\mu_{r,1}$), and the relative permittivity and permeability of the wall ($\varepsilon_{r,2}$ and $\mu_{r,2}$). The material properties determine the field impedance $Z_{\mathrm{field}}$ and refractive index *n* of the medium.

(17) $$\begin{array}{c} Z_{\mathrm{field}}=120\pi \sqrt{\frac{\mu_{r}}{\varepsilon_{r}}} \end{array}$$

(18) $$\Gamma_{⊥}=\frac{Z_{2}\cos\left(\phi_{i}\right)-Z_{1}\sqrt{1-{\left(\frac{Z_{2}}{Z_{1}}\right)}^{2}\sin{\left(\phi_{i}\right)}^{2}}}{Z_{2}\cos\left(\phi_{i}\right)+Z_{1}\sqrt{1-{\left(\frac{Z_{2}}{Z_{1}}\right)}^{2}\sin{\left(\phi_{i}\right)}^{2}}}$$

The resulting reflection coefficient $\Gamma_{⊥}$ represents a ratio of electric field strengths. ${\left|\Gamma \right|}^{2}$ is used for the calculation of the reflection power loss, which is a ratio of Poynting vectors (as explained in [54]). Equation (19) expresses the reflection power loss $L_{r}$ in dB, assuming only transverse electric reflections.

(19) $$L_{r}\left(\mathrm{dB}\right)=P_{i}\left(\mathrm{dBm}\right)-P_{r}\left(\mathrm{dBm}\right)=-10\log_{10}{\left|\Gamma_{⊥}\right|}^{2}$$

Besides the specular reflection, signal transmission and refraction occurs at material boundaries, as illustrated in Figure 4. In case of homogenous walls, the transmitted ray can result in a (weak) contribution to the specular reflection. In this case, multiple reflection and transmission coefficients should be included, as well as absorption. Signal absorption for brick walls has been reported as 42 dB/m at 2.4 GHz [55,56]. However, walls can mostly not be considered as homogenous structures because of cavities, metal structures for reinforcement or support, or other imperfections. These inner structures are generally unknown and make transmitted rays highly unpredictable and thus invaluable (cfr. scattered and diffracted rays). Because of the reduced signal strength and unpredictability of transmitted rays, these components are not considered for ray tracing. This approach is called a ‘thin wall’ approximation, which was validated in [48].

For each simulated ray, the loss $L_{\mathrm{ray}}$ is calculated as a sum of the free space path loss ($L_{\mathrm{path},\mathrm{free}}$) and all reflection losses against north-south oriented walls ($L_{r,NS}$) and east-west oriented walls ($L_{r,EW}$) [50]. $L_{\mathrm{path},\mathrm{free}}$ is calculated according to the Friis path loss Equation (20) with free space path loss exponent $n_{\mathrm{path}}=2$ and *d* representing the unfolded path length of the ray [57]. The $L_{r,NS}$ and $L_{r,EW}$ values are calculated according to Equations (18) and (19).

(20) $$L_{\mathrm{path}}=-10\cdot \log_{10}{\left(\frac{\lambda }{4\pi d}\right)}^{n_{\mathrm{path}}}$$

(21) $$L_{\mathrm{ray}}\left(\mathrm{dB}\right)=L_{\mathrm{path},\mathrm{free}}+\sum L_{r,NS}+\sum L_{r,EW}$$

The execution of the ray tracing algorithm requires knowledge of the relative permittivity (i.e., the dielectric constant) of the walls. Therefore, a literature study was performed focusing on material properties in the 2.4 GHz ISM band. The reported permittivity of bricks showed to be relatively consistent, with the relative permittivity ranging from 3.82 to 4.75 [56,58,59,60,61].

**Figure 5:**
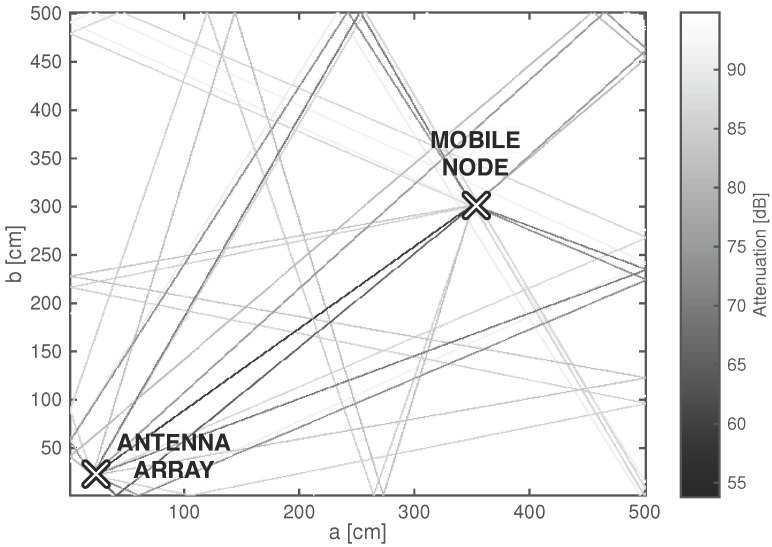
Simulated rays in a room of 5 m by 5 m with brick walls.

The result is a 2D ray tracer, simulating LOS signals and specular reflections for multipath assisted localization. An example of a simulated 5 m by 5 m room with brick walls is presented in Figure 5. The simulation of this room with fourth order reflections on a 2.3 GHz Intel^®^ Core i5 2415M mobile CPU with 8 GB of RAM, takes 0.17 s.

### 3.5. Reference Data

The fingerprint vectors $f_{i}$ that are gathered in multipath simulations are matched to a measurement vector m to find the location of a node. m represents the measured AoA data, which consists of a spatial spectrum $m_{\mathrm{MVDR}}$ (or a discrete angular value $m_{\mathrm{LOS}}$). A similar format is desired for the fingerprint vectors. Therefore, each fingerprint vector $f_{i}$ consists of a spatial spectrum $P_{\mathrm{sim}}\left(\theta \right)$, which is generated by the multipath simulator. The result is a training data set T, consisting of simulated spatial spectra. The fingerprint vectors are only used for their AoA data and not for the overall signal power. Therefore, the simulated spatial spectra $P_{\mathrm{sim}}\left(\theta \right)$ [dB] are normalized to angular probability density functions $P_{\mathrm{PDF},\mathrm{sim}}\left(\theta \right)$, as described by Equation (22) and illustrated in the following sections. As a result, all fingerprints $f_{i}$ have the same overall weight $\left(\int f_{i}d\theta =1\right)$, possibly simplifying matching algorithms.

(22) $$P_{\mathrm{PDF},\mathrm{sim}}\left(\theta \right)=\frac{P_{\mathrm{sim}}\left(\theta \right)-\min\left[P_{\mathrm{sim}}\left(\theta \right)\right]}{\int_{-90^{\circ }}^{90^{\circ }}P_{\mathrm{sim}}\left(\theta \right)-\min\left[P_{\mathrm{sim}}\left(\theta \right)\right]d\theta }$$

#### 3.5.1. Artificial Spatial Spectrum Based on Ray Tracing

**Figure 6:**
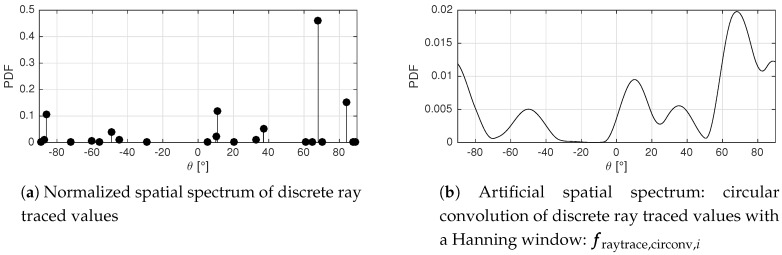
Fingerprint examples.

For the generation of reference data, the discrete outputs of the ray tracing algorithm are used. For each simulated ray, the signal attenuation $L_{\mathrm{ray}}$ and the AoA $\theta_{\mathrm{ray}}$ is available. Plotting the signal attenuations for all simulated rays with $\theta \in [-90^{\circ },90^{\circ }]$ results in a simulated spatial spectrum with discrete peaks, as demonstrated in Figure 6 which depicts a normalized discrete spectrum.

However, the goal is to achieve a result that resembles a measured MVDR spatial spectrum. In order to create the artificial spatial spectrum, the discrete ray tracing spectrum is circularly convolved with a filter window h(θ), as expressed in Equation (24). For the calculation of the circularly convolved spectrum $P_{\mathrm{PDF},\mathrm{circonv}}\left(\theta \right)$, a periodic discrete spectrum is defined: $P_{\mathrm{PDF},\mathrm{raytrace},\mathrm{discrete},T}\left(\theta \right)$.

(23) $$P_{\mathrm{PDF},\mathrm{discrete},T}\left(\theta -90^{\circ }+k\cdot 180^{\circ }\right)=P_{\mathrm{PDF},\mathrm{discrete}}\left(\theta -90^{\circ }\right)$$

(24) $$P_{\mathrm{PDF},\mathrm{circonv}}\left(\theta \right)={\left[\left(P_{\mathrm{PDF},\mathrm{discrete},T}*h\right)\left(\theta \right)\right]}_{\theta \in [-90^{\circ },90^{\circ }]}$$

Because the MVDR spatial filter weights $w_{MVDR}$ depend on the received data, the filter shape and bandwidth are variable and difficult to determine [42]. Therefore, the shape of the filter window h(θ) of the artificial spectrum was empirically chosen. A Hanning window was selected for its limited side lobes and zeros at the end points of the window, preventing discontinuities after the convolution. The width of the window was empirically fixed as $W=180^{\circ }/\left(M-K_{ss}-1\right)$, following Equation (15). Figure 6 depicts an artificial spatial spectrum $f_{\mathrm{raytrace},\mathrm{circonv},i}$, which was created with the proposed method.

(25) $$h\left(\theta \right)=\frac{1}{2}{\left(1-\cos\left(\frac{360^{\circ }\theta }{W}\right)\right)}_{\theta \in [0,W]}$$

#### 3.5.2. LOS Reference-Benchmark

**Figure 7:**
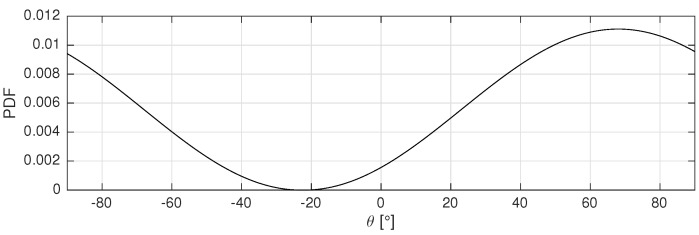
LOS fingerprint example: $f_{\mathrm{LOS},i}$ for $\theta_{\mathrm{LOS}}=68^{\circ }$.

In order to assess the performance of the proposed multipath assisted methods, a benchmark localization approach is used. For fair comparison, this standard method relies on the same fingerprint based localization framework with $f_{i}$ and m vectors, but only LOS directions are considered. The fingerprint vectors $f_{\mathrm{LOS},i}$ are given by the $P_{\mathrm{sim},\mathrm{LOS}}\left(\theta \right)$ spatial spectra, as represented in Equation (26) and depicted in Figure 7. This function can be considered as the circular convolution of a discrete LOS peak with a $180^{\circ }$ wide Hanning window.

(26) $$f_{\mathrm{LOS},i}=P_{\mathrm{sim},\mathrm{LOS}}\left(\theta \right)=\frac{1}{2}{\left(1+\cos\left(\frac{\theta -\theta_{\mathrm{LOS}}}{2}\right)\right)}_{\theta \in [-90^{\circ },90^{\circ }]}$$

### 3.6. Matching Algorithm

As described in literature, the kNN algorithm is an established method for position estimation in a fingerprinting system [9,33]. This section describes a similar approach, tailored for the proposed localization framework. The kNN method performs weighted averaging of *k* position estimates in order to achieve a higher resolution than the coarse training grid. However, the proposed multipath assisted system uses simulated training data with a much finer grid than survey based systems. Therefore, the averaging step is redundant, resulting in a 1NN approach. In order to find the nearest neighbor, the match between the measurement vector m and each fingerprint $f_{i}$ is rated. The Pearson correlation coefficient $r_{\mathrm{corr}}\left(i\right)$ can be used as a means for rating the resemblance between m and $f_{i}$, as proposed in [62]. The absolute signal strength does not affect these values, since only the shape of the curves is considered.

(27) $$\begin{array}{cc} r_{\mathrm{corr}}\left(i\right) & =\frac{\mathrm{cov}(f_{i},m)}{\sqrt{\mathrm{var}\left(f_{i}\right)\cdot \mathrm{var}\left(m\right)}} \end{array}$$

The obtained coefficients $r_{\mathrm{corr}}\left(i\right)$ can be scaled linearly to a Spatial Probability Density Function (SPDF) $r_{\mathrm{SPDF},\mathrm{corr}}\left(i\right)$, representing the probability for each position $p_{i}$.

(28) $$r_{\mathrm{SPDF},\mathrm{corr}}\left(i\right)=\frac{r_{\mathrm{corr}}\left(i\right)+1}{N_{f}+\sum_{i=1}^{N_{f}}r_{\mathrm{corr}}\left(i\right)}$$

**Figure 8:**
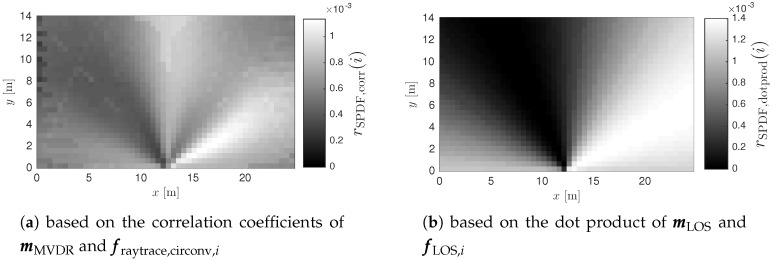
Example surface probability density functions.

The position estimate $\tilde{p}=p_{j}$ is determined by the highest value of the SPDF. However, it should be noted that a LOS benchmark SPDF for a single antenna array cannot be used for location estimation, as only one angular component is measured. The resulting SPDF represents a line or beam, as illustrated in Figure 8.

(29) $$j=\arg\max_{i\in \left\{1,\ldots ,N_{f}\right\}}r_{\mathrm{SPDF}}\left(i\right)$$

### 3.7. Multi-Anchor Configurations

**Figure 9:**
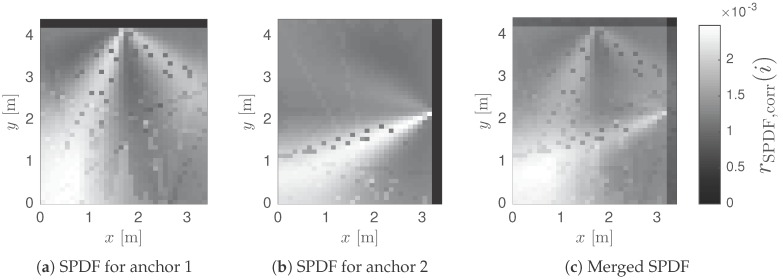
Example of two merged SPDF vectors.

When *B* antenna arrays are placed in a room, a set of training data $T_{b}$ is generated for each array ($b\in \left\{1,\ldots ,B\right\}$). An SPDF $r_{\mathrm{SPDF},b}\left(i\right)$ is calculated for each array, based on $m_{b}$ and $f_{b,i}$. When all SPDF vectors are equally sized, they can be linearly combined with equal weights, as expressed by Equation (30). Figure 9 illustrates how the results of two anchor nodes can be merged to a single SPDF vector to obtain a more confined location estimate.

(30) $$r_{\mathrm{SPDF}}\left(i\right)=\frac{1}{B}\sum_{b=1}^{B}r_{\mathrm{SPDF},b}\left(i\right)$$

### 3.8. Localization Error

**Figure 10:**
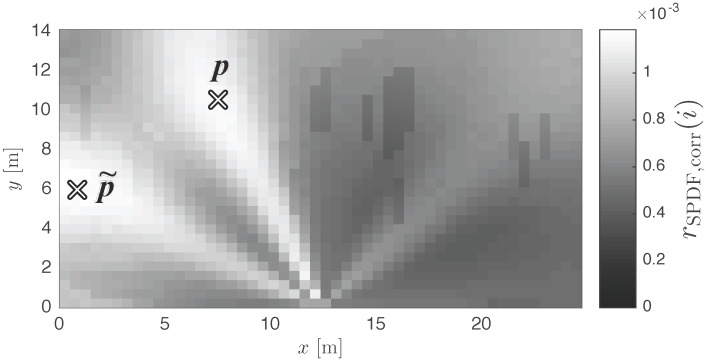
SPDF example for a NLOS measurement, resulting in a large localization error $\epsilon_{\mathrm{loc}}$ and a small surface interval.

The most straightforward measure to evaluate the performance of a localization system or algorithm is the localization error $\epsilon_{\mathrm{loc}}$. This quantity is defined as the euclidean distance between the estimated position $\tilde{p}$ and the real position p of the mobile node. In order to compare localization errors in differently sized environments, the error can be normalized to the diagonal of the testing area, resulting in ${\hat{\epsilon }}_{\mathrm{loc}}$. Many localization algorithms converge to a single location estimate, making the localization error an adequate criterion for the assessment of the system performance. However, the proposed multipath assisted localization technique is not a finalized system and intermediate results do not necessarily converge to a single position. Figure 10, depicts an SPDF for a NLOS measurement, converging to two positions. The wrong one is selected as position estimate $\tilde{p}$, resulting in a large localization error, undervaluing the performance of the algorithm. In this case, the algorithm should still be considered very valuable, as auxiliary methods can easily result in a very accurate location estimate (e.g., dead reckoning techniques, an extra anchor node, etc.).

Another shortcoming of localization errors is related to the benchmark algorithms. As explained in Section 3.6, no location $\tilde{p}$ can be estimated in a single anchor node system with the LOS benchmark algorithm. As such, it is impossible to calculate a $\epsilon_{\mathrm{loc}}$ value for the LOS benchmark method.

### 3.9. Surface Interval

In order to overcome the $\epsilon_{\mathrm{loc}}$ related problems, a new measure is proposed for rating the accuracy of the system. The ‘Surface Interval’ (SI) is a dimensionless quantity between 0 and 1, representing the percentile of the SPDF that contains the real position p, as expressed in Equation (31). Hence, SI indicates the fraction of the room surface that should be isolated to contain p. Obviously, an SI value close to zero represents a high accuracy.

(31) $$S\;I=P\left(r_{\mathrm{SPDF}}\left(i\right)>r_{\mathrm{SPDF}}\left(p\right)\right)$$

#### Overall System Accuracy

The ${\hat{\epsilon }}_{\mathrm{loc}}$ and SI parameters provide information on a single position estimation. However, for an overall accuracy assessment in a certain environment, multiple localization tests are performed along a uniformly distributed grid of mobile node positions in the room. A Cumulative Density Function (CDF) could be used for the representation of ${\hat{\epsilon }}_{\mathrm{loc}}$ and SI values. However, when a large number of configurations is evaluated, the CDF approach is inadequate. Therefore, the 50th percentile P50 (i.e., the median value), the 95th percentile P95, and the mean value of ${\hat{\epsilon }}_{\mathrm{loc}}$ and SI are listed, forming an ideal tool for the evaluation and comparison of system accuracies. Furthermore, the P50 and P95 surface intervals indicate the effectiveness of the algorithm: a P50=0.50 or P95=0.95 value indicates a completely random localization algorithm. In another respect, if 50% (or 95%) of the SPDF should be selected for a 50% (or 95%) probability of including position p, the system can be considered useless. The localization errors ${\hat{\epsilon }}_{\mathrm{loc}}$ are not omitted in the evaluations because they still provide the most tangible measure of localization accuracy. Also they enable the comparison to localization systems in literature. Throughout this work, the ‘accuracy’ or ‘performance’ of localization algorithms form a general reference to the ${\hat{\epsilon }}_{\mathrm{loc}}$ and SI parameters. The ‘robustness’ of a system denotes the immunity to adverse influences (e.g., NLOS connections).

## 4. Experimental Results

**Figure 11:**
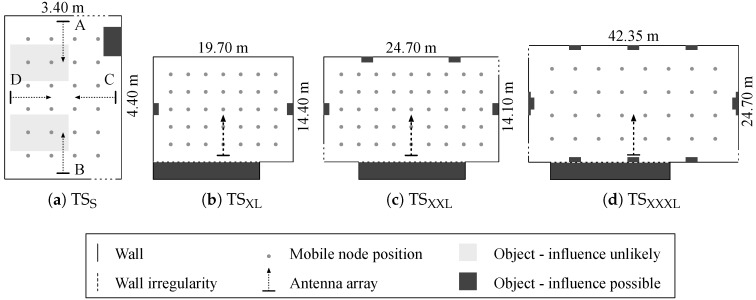
Overview of the test environments.

In order to test, evaluate and configure the localization system, measurements are performed in various real-world environments. Rectangular rooms are selected and subdivided by measurement grids, uniformly distributing test positions of the mobile node. Figure 11 depicts the floor plans of all test setups, including the measurement grid, array positions and objects in the room. One small-sized room was considered with multiple array positions: ${\mathrm{TS}}_{S}$. Also, three larger test setups (sports halls) were considered with a large number of test points: ${\mathrm{TS}}_{\mathrm{XL}}$, ${\mathrm{TS}}_{\mathrm{XXL}}$ and ${\mathrm{TS}}_{\mathrm{XXXL}}$.

The antennas are always placed in the same horizontal plane at a height of 1.3 m, resulting in $0^{\circ }$ elevation angles. Since ray tracing simulations only account for the walls of a rectangular room, the objects are not considered in the reference data, however they can have an influence on system accuracy. When obstacles are significantly lower than the antenna heights and feature a limited amount of metallic parts (e.g., tables), these objects are not expected to strongly interfere with the simulated multipath and can therefore be classified as ‘unlikely influential’. Large objects and metal structures at antenna heights are classified as ‘possibly influential’.

- ${\mathrm{TS}}_{S}$: A 4×6 measurement grid is established. The reference set contains a finer 0.11 m grid of 30×40 positions. Four array positions are considered: in the middle of each wall (A–D) (simulation time approximately 204 s).
- ${\mathrm{TS}}_{\mathrm{XL}}$: The smallest sports hall contains a 7×5 measurement grid and a 40×30 reference grid (simulation time approximately 204 s).
- ${\mathrm{TS}}_{\mathrm{XXL}}$: A 9×5 measurement grid is used in this setup, with a reference grid of 50×30 (simulation time approximately 255 s).
- ${\mathrm{TS}}_{\mathrm{XXXL}}$: The measurement grid contains 8×4 positions and 84×50 reference grid points were simulated (simulation time approximately 714 s).

In all setups, measurements are performed at 2.47 GHz with a 10-element λ/2 array configuration, as detailed in [63]. For each position of the mobile node, a LOS and NLOS measurement is performed. In NLOS conditions, the LOS signal is blocked with Eccosorb VHP-8 absorbers [64], attenuating the LOS component with at least 20 dB. In the small test setups, a single 0.6 m by 0.6 m absorber tile was used, while the larger rooms admitted a 1.2 m by 1.2 m absorber. The absorbing tiles are always placed between the transmitting and receiving antenna, blocking a $20^{\circ }$ to $45^{\circ }$ field of the omnidirectional mobile transmitter.

In literature, NLOS localization tests generally focus on static NLOS situations [65,66,67], with experiments behind corners in complex indoor environments. Because these infrastructures are static, the NLOS channel characteristics can be fully known and exploited. In contrast, the new approach in this research emulates a dynamic situation where LOS conditions can change to NLOS. This mimics real-world situations where moving people or furniture (temporarily) obstruct LOS connections. These environmental changes are unknown to the positioning system, so all localization tests are performed with the same reference data set T, assuming LOS conditions. This reproducible approach enables a straight-forward comparison between LOS and NLOS results.

### 4.1. Localization Performance as a Function of the Array Size

All AoA evaluations are performed with a 10-elements array, which is the largest configuration that can be formed with the available setup at 2.47 GHz with λ/2 inter-element spacing. This section discusses the accuracy of the indoor localization system as a function of the number of array elements. Furthermore, the relationship between the number of antennas, spatial smoothing and the localization accuracy is investigated. For these evaluations, the results of ${\mathrm{TS}}_{\mathrm{XL}}$, ${\mathrm{TS}}_{\mathrm{XXL}}$ and ${\mathrm{TS}}_{\mathrm{XXXL}}$ are merged, resulting in a data set of 122 positions to be localized. Evaluations are performed in LOS and NLOS conditions for both the benchmark algorithm and the multipath assisted algorithm. All results are based on the same phase and amplitude measurements of a 10-element array, from which channels are eliminated to evaluate the performance with less antenna elements.

**Figure 12:**
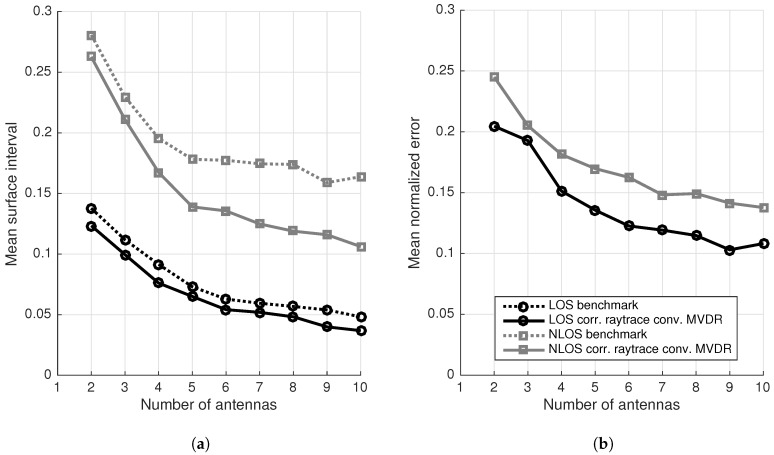
Evaluation of the mean surface interval (**a**) and mean normalized errors (**b**) as a function of the number of array elements (*M*).

**Figure 13:**
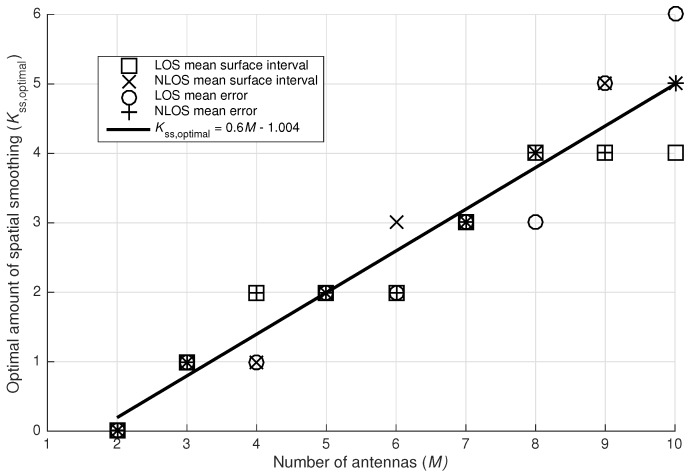
Optimal amount of spatial smoothing.

Figure 12a,b depict respectively the mean surface interval and mean normalized error as a function of the number of array elements. These results heavily depend on the applied amount of spatial smoothing. This parameter is depicted in Figure 13. In Figure 12a,b only the best achievable results are presented. This means that for each point in these graphs, only the best result of all possible spatial smoothings is depicted.

Figure 12a clearly illustrates how the proposed localization algorithm outperforms the standard benchmark algorithm in LOS and NLOS situations for any number of array elements. This effect is manifested most clearly in NLOS situations when the number of antennas increases, providing more multipath information. As soon as four antennas are used, the proposed algorithm performs equally or better than the benchmark algorithm that uses one more antenna in NLOS and LOS situations. For localization systems with the benchmark approach and five or more antennas, this means that the array size can be cut without reducing the accuracy of the system, just by using the proposed localization algorithm. With five antennas, the proposed algorithm outperforms the benchmark algorithm in all NLOS configurations till 10 elements. Figure 12b confirms the previous conclusions. Logically, an increase in array size results in a reduction of the localization errors. The graph also illustrates that LOS accuracies can be achieved in NLOS situations, if more antennas are added. This is also illustrated in Figure 12a, however this holds only for the proposed algorithm, which takes multipath components into account.

As already mentioned, the graphs in Figure 12a,b only depict the best results of all possible spatial smoothings. Figure 13 indicates the optimal amount of spatial smoothing $K_{\mathrm{ss},\mathrm{optimal}}$ as a function of the number of antennas *M*. The optimization of spatial smoothing is a minimization of the mean surface intervals and the mean normalized localization errors in LOS and NLOS situations. The discrete points can be approximated with a linear regression. The result of such a least-squares linear regression is expressed in Equation (32), taking all discrete points into account. Of course, only a discrete number of spatial smoothings can be performed, so if the equation is used for determining $K_{\mathrm{ss},\mathrm{optimal}}$ in a given setup, a rounded value should be used. Normally, the point (2,0) should be part of the curve, as spatial smoothing is impossible in 2-element arrays, following Equation (15). After rounding, the correct value is obtained.

(32) $$K_{\mathrm{ss},\mathrm{optimal}}\left(M\right)=0.600\cdot M-1.004$$

### 4.2. Antenna Distribution

This paragraph investigates how the accuracy can be enhanced with restrictions on the cost of the system and complexity of hardware. More specifically, experiments are performed in the ${\mathrm{TS}}_{S}$ setup with one 10-element array (positions A and B), which is compared to a setup with two 5-element ULAs, located on neighboring walls (AC, AD, BC, BD). This solution requires the same amount of receiver channels, however increased performance could be achieved due to their spatial separation, similar to distributed antenna communication systems [68]. For the 5-element and 10-element arrays, respectively two and five spatial smoothings were applied, following the results of Section 4.1.

Table 1 presents the outcome of this experiment. The results clearly indicate that two 5-element arrays offer a significantly higher localization accuracy over a single 10-element array. This effect is the strongest in LOS conditions, with halved localization errors and strongly decreased surface intervals. In (dual) NLOS conditions the same conclusions hold, yet less pronounced: mean normalized localization errors are improved from 14.5% to 10.6%, but surface intervals remain inconclusive due to contradictory mean and median values.

The results in Table 1 also confirm the value of multipath information, as a 10-element array clearly benefits from the optimized localization algorithm. This is most prominently illustrated by the NLOS surface intervals. In 5-element arrays the achievable improvement from multipath information is less explicit, as only two signal components can be distinguished with the given amount of spatial smoothing. As a conclusion, it is fair to state that multiple small spatially distributed arrays offer a more robust solution than setups that rely on one large array. When a quick and less intrusive installation is desired, a system with a single large array can be applied in combination with the optimized localization algorithm.

### 4.3. Multiple Arrays

Previous paragraphs focused on the use of a single antenna array or dual arrays for localization purposes in rectangular rooms. However, localization accuracy can be further increased by adding more arrays to the room. This results in more spatial information and increases the chance on receiving LOS signals. The following tests are performed in the environment ${\mathrm{TS}}_{S}$ with maximum four antenna arrays (positions A, B, C and D). This section compares the performance of systems with one to four 10-element antenna arrays in all possible combinations of LOS and NLOS connections. In one-array setups, the array is placed against a shorter wall (A or B). In 2-array setups only neighboring arrays are considered (AC, AD, BC and BD). For 3-array setups, all possible 3-array configurations are included (ACB, BDA, CAD and DBC). In 4-array tests, only one configuration remains: ABCD. For each system, the influence of NLOS connections is investigated by gradually increasing the number of NLOS connections from zero to maximum.

**Figure 14:**
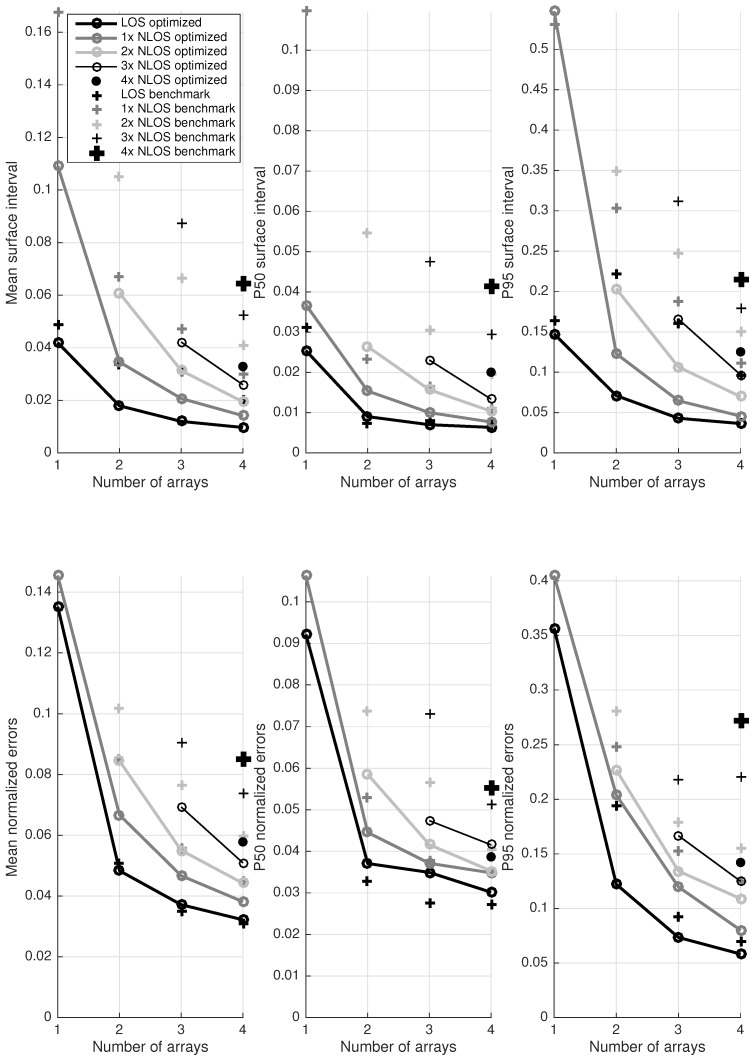
Evaluation of system performance for multiple antenna arrays with all-LOS to all-NLOS connections.

Figure 14 provides an overview of all test results. Mean, P50 and P95 values of surface intervals and normalized localization errors are presented in six separate graphs. Each graph contains results for the benchmark algorithm (plus-signs) and the optimized algorithm (dots, connected by a line). The lines interconnect the results with an equal number of NLOS connections: zero (i.e., only LOS connections) to four. The discussion of these graphs is split into three parts, treating the influence of the number of arrays, (N)LOS connections, and a comparison between the optimized and benchmark algorithm. In the discussions, a situation with only LOS or only NLOS connections is called respectively an all-LOS or all-NLOS situation.

#### 4.3.1. Number of Arrays

The results of the optimized localization algorithms can be observed as a function of the number of arrays, providing some insight in the expected accuracy of different setups. In the next analysis, we take all results into account, including the worst all-NLOS configurations. One-array systems clearly exhibit the poorest performance in terms of surface intervals and localization errors. Mean localization errors amount 14% of the room diagonal (0.78 m in the considered setup), giving a general estimation of the location. The potential performance of the algorithms is illustrated by P50 surface intervals under 4%. However, the one-array setup is not highly reliable with P95 localization errors of 40% of the room diagonal (2.22 m in the considered setup) and P95 surface intervals up to 54%.

Increasing the number of antenna arrays vastly improves performance. Adding just a second array almost halves the P95 values of normalized localization errors to 23% and mean values stay below 9% (0.50 m in the setup). As more arrays are added to the system, further accuracy improvements can be noticed, however the rate of improvement decreases with more arrays. The 4-array setup represents a very accurate and reliable system, which is demonstrated by surface intervals and localization errors. P95 surface intervals stay below 13% and P95 normalized localization errors do not exceed 15%. The median localization error for this setup never exceeds 4.2% of the room diagonal (0.23 m). In a real-time tracking implementation, an even higher performance is expected, as 4xNLOS situations are unlikely and post-processing techniques (e.g., dead reckoning, particle filters, Kalman filters, etc.) can be applied, depending on the application.

#### 4.3.2. NLOS Connections

Intuitively, LOS situations can be expected to yield the best results. This statement can be underpinned with an analysis of LOS and NLOS connections in Figure 14. The graphs clearly illustrate that an all-LOS situation always performs best. As soon as two arrays are used, good results are obtained in LOS conditions. Adding more arrays is mainly useful to account for NLOS connections. When two LOS connections are available, mean normalized localization errors under 5% can be expected, as well as P95 values under 12% (0.67 m in the setup). An important remark is that having an additional array with a NLOS connection is always better than having no additional array at all. So generally, NLOS connections still provide useful information that increases the accuracy instead of deteriorating system performance.

All-NLOS scenarios clearly influence surface intervals, with mean values tripling in comparison to the all-LOS scenario. Also in localization errors, an obvious influence can be remarked. The only solution for maximizing the accuracy in an all-NLOS scenario consists of using as much arrays as possible. With four arrays, it is possible to achieve P95 localization errors below 14% (0.83 m in the setup).

#### 4.3.3. Optimized vs. Benchmark Algorithm

Previous discussions of Figure 14 only considered the results of the optimized localization algorithm. The benchmark results are also depicted, enabling an interesting assessment of the new algorithm in comparison to the benchmark. The figure shows that the new algorithm outperforms the benchmark in surface intervals and localization errors (with a specific exception of all-LOS P50 localization errors). Benchmark algorithms regularly exhibit double surface interval values, demonstrating their inferior performance. In localization errors, the differences are sligthly less explicit, but they lead to the same conclusion: taking multipath effects into account leads to more accuracy than the classical AoA approach. More specifically, the proposed localization algorithm can achieve similar or better results with less antenna arrays. In several cases, a two-array system with the new algorithm performs better than a 4-array approach with the conventional algorithms, possibly halving hardware and installation costs. Examples of this statement can be seen in all-NLOS localization errors.

In a 4-array system with all-NLOS connections and the benchmark algorithm, P95 normalized localization errors of 27% can be observed. This 1.50 m P95 uncertainty in a 3.4 m × 4.4 m room can be considered unsatisfactory, given the expensive setup of a 4-array localization system. This result also demonstrates the unsuitability of the benchmark algorithm for NLOS localization.

## 5. Discussion

The proposed localization technique was evaluated in a variety of real-world environments and the results can be compared to the related work that was presented in Section 1. Our research results were considered comparable to literature, as all papers describe experimental setups, demonstrating localization techniques with a single mobile node. Although further research is required to obtain a practically deployable real-time multi-node localization system, this assessment provides useful insights in the performance of the developed positioning techniques.

In [69], a mean normalized error of 6.1% is reported for the standard triangulation approach with three arrays in a LOS area. For our system, a value of 3.8% is achieved in these ideal conditions, an improvement that can be attributed to superior hardware (e.g., more antennas). The single anchor AoA fingerprinting systems of [29,30,31] exhibit mean LOS normalized errors between 21.6% and 29.2%. Our approach generally scores between 10% and 15% depending on the size of the array, which illustrates the superior accuracy of the proposed system over a labor intensive fingerprinting implementation. In NLOS conditions, this fingerprinting approach yields mean normalized errors of 26.8%, compared to 14% to 18% values for the proposed system. A reason can be found in the fine resolution of the calculated reference set. Also, these calculations only take LOS and specular components into account according to their expected signal strengths. This appears to be a more accurate solution than relying on a single snapshot of the multipath environment. Especially in NLOS conditions, multipath calculations outperform fingerprinting. Some fingerprinting systems in literature provide a higher localization accuracy in LOS conditions [21,32,66]. However, these implementations rely on multiple anchor nodes or even an antenna array at the mobile node. Furthermore, these systems are not tested in a adverse (NLOS) conditions.

The multipath assisted UWB systems that are presented in [24,25,26,27,28,67] deliver another class of performance. With P95 values of normalized errors below 3%, these systems can be considered extremely accurate and reliable, compared to the 36% value for our single anchor system. These exceptional results can be attributed to the UWB ToA approach, delivering an inherently higher accuracy in comparison to narrowband systems. Only [67] considered NLOS conditions. This publication reported median normalized errors of 6.3%, still exceeding the LOS performance of our approach. However, UWB systems should not be considered as a better alternative in all situations. While narrowband AoA hardware can be found in contemporary communication systems, UWB localization systems rely on dedicated and costly infrastructure.

The proposed localization approach does not only result in an increased localization accuracy with respect to standard AoA or single-anchor systems, but also reduces the setup efforts in comparison to conventional fingerprinting methods. Because our proposed technique relies on simulated fingerprints no labor intensive site surveying is required, so this method can be used in an easily deployable indoor positioning system (EDIPS). As stated in Section 4, offline multipath calculations take between 3 and 12 min for the considered rooms. Furthermore, the technique was developed with contemporary communication systems in mind, bringing the deployment efforts at the same level as EDIPS in literature [34,35,36]. However, these papers reported coarse (room-level) accuracy, while the new multipath assisted approach even allows sub-meter accuracy (depending on the setup).

## 6. Conclusions and Future Work

In this work, a new AoA localization approach is proposed. With this technique, an increased accuracy and reduced setup effort is aspired. This research only focused on the experimental validation of the proposed algorithm, rather than the development of a multi-user real-time localization system. The proposed positioning method relies on an AoA measurement vector m, which is matched to a set of simulated reference vectors $f_{i}$, resembling a fingerprinting approach. The measurement vector consists of an MVDR spatial spectrum, indicating the incident power for all angles. Forward-backward averaging and spatial smoothing were applied as pre-processing techniques. The reference vectors are computed in a multipath simulation framework, which relies on a-priori known floor plan information. Therefore, a 2D ray tracer was developed, simulating LOS and specularly reflected signal components according to the image method. The results can be represented as an artificial spatial spectrum, created as a circular convolution of discrete ray tracing data with a Hanning window. The matching of a measurement vector with reference vectors is performed by calculating correlation coefficients. These values result in an SPDF, indicating the location probability.

The indoor positioning technique was experimentally tested in four real-world environments. An evaluation of hardware configurations demonstrated that the accuracy increases as the number of antennas varies from 2 to 10. Also the optimal amount of spatial smoothing was defined as a function of the number of antennas in the array ($K_{\mathrm{ss}}=5$ for M=10). Furthermore, the spatial distribution of antennas was investigated, revealing that two 5-element arrays deliver a higher accuracy than one 10-element array. The localization accuracy was studied for systems with up to four arrays and all possible combinations of LOS and NLOS connections. These results indicated that the multipath assisted approach can result in similar or better performance than the benchmark algorithm, while using less anchor nodes. Especially in NLOS situations, the proposed method delivers a significant accuracy improvement.

The overall accuracy of the multipath assisted AoA positioning method cannot be reduced to a single value due to the variety of system configurations, however some examples can illustrate the achievable accuracy. A single 10-antenna anchor node results in median normalized errors around 9% in LOS conditions, while NLOS situations yield values around 11%. In 4-anchor setups, these results improve to 3% for LOS, and 4% in NLOS situations. The exact configuration of the localization system depends on the required accuracy and robustness. The proposed technique exhibits significant performance improvements over the benchmark algorithms and comparable systems in literature, especially in NLOS conditions. However, the exceptional performance of dedicated UWB multipath assisted solutions was not matched.

The presented research provides a proof of concept for indoor multipath assisted AoA positioning, relying on narrowband signals and antenna arrays. These properties make the technology compatible with emerging MIMO or MaMIMO communication systems, allowing a ‘signals-of-opportunity’ approach for the localization system. The proposed method uses multipath propagation as a valuable source of information, even in NLOS conditions. This results in an increased accuracy and robustness, or a reduction of system complexity and cost. Furthermore, the calculation of multipath fingerprints in computer simulations allows a serious reduction of setup efforts, allowing quick and easy deployment.

Since this research only provides a proof-of-concept of the multipath assisted AoA localization technique, a logical evolution is the development of a real-time multi-user indoor localization system. Furthermore, the system can be merged with contemporary communication systems, similar to the way that RSS localization is currently implemented in WiFi systems. A concrete application is envisioned in future 5G cellular networks, which aim for localization and communication in a single MaMIMO framework. However, any other MIMO communication technology is a potential candidate.

In order to come to a commercially deployable system, further hardware and software development is required. At the hardware side, different array structures can improve localization accuracy and reduce hardware complexity. Further improvements of the localization algorithm are aimed at the practical applicability in real-world situations: a deployable system should allow arbitrarily shaped room layouts, which require a more advanced multipath simulator. Furthermore, an important feature that should be implemented, is real-time travelled path tracking of multiple moving transmitters. In this context, further accuracy improvements can be achieved with dead reckoning techniques, Kalman filtering or particle filters. In the context of multi-user localization, the communication infrastructure can assist in the identification of different nodes.

## Figures and Tables

**Table 1:** Comparison of one 10-element array with two 5-element arrays in test setup ${\mathrm{TS}}_{\mathrm{brick}}.$

			Surface Interval			Normalized Error		
	**Setup**	**Algorithm**	**Mean**	**P50**	**P95**	**Mean**	**P50**	**P95**
LOS	1×10	benchmark	0.049	0.031	0.163	−	−	−
		optimal	0.042	0.025	0.148	0.135	0.092	0.357
	2×5	benchmark	0.040	0.010	0.220	0.075	0.043	0.289
		optimal	0.025	0.013	0.089	0.061	0.045	0.162
NLOS	1×10	benchmark	0.168	0.110	0.531	−	−	−
		optimal	0.110	0.037	0.547	0.145	0.106	0.405
	2×5	benchmark	0.125	0.067	0.432	0.116	0.086	0.269
		optimal	0.094	0.055	0.280	0.106	0.095	0.270

## Abbreviations

The following symbols are used in this manuscript:

Γ	Reflection coefficient
$\epsilon_{\mathrm{loc}}$	Localization error
$\varepsilon_{r}$	Relative permittivity
Δ [m]	Inter element distance
Δh [m]	Boundary imperfection
η(t)	Noise signal
θ	Angle of arrival
λ	Wavelength
$\mu_{l}$	Phase difference between array elements
$\mu_{r}$	Relative permeability
$\Pi_{M}$	M×M exchange matrix
$\sigma_{\eta }^{2}$	Noise variance
τ	Time delay
$\phi_{i}$	Angle of an incident wave
$\phi_{r}$	Angle of a reflected wave
$\phi_{t}$	Angle of a transmitted wave
a(θ)	Array steering vector
A	Array manifold
*B*	Number of antenna arrays
*c*	Speed of light
*d*	Distance
E	Electric field vector
*f*	Frequency
f	Fingerprint vector
H	Magnetic field vector
H(f,θ)	Antenna response
h(θ)	Filter window
$K_{ss}$	Number of spatial smoothings
*L* [dB]	Loss
*L*	Number of signal sources
m	Measurement vector
*M*	Number of antennas in an array
*n*	Refractive index
*N*	Number of array snapshots
p	Position vector
P(θ)	Spatial spectrum
$P_{i}$	Power of an incident wave
$P_{r}$	Power of a reflected wave
$P_{t}$	Power of a transmitted wave
$P_{\mathrm{PDF}}\left(\theta \right)$	Angular probability density function
R	Spatial covariance matrix
$R_{\eta }$	Spatial correlation matrix of η(t)
r(t)	Received signal
$r_{\mathrm{corr}}$	Pearson correlation coefficient
$r_{\mathrm{SPDF}}$	Spatial probability density function
S	Spatial correlation matrix of s(t)
s(t)	Transmitted signal
SI	Surface interval
T	Training vector
w	Weight vector
*W*	Window width
*Z*	Impedance
